# Single Mothers by Choice: Parenting and Child Adjustment in Middle Childhood

**DOI:** 10.1037/fam0000797

**Published:** 2020-09-17

**Authors:** Susan Golombok, Sophie Zadeh, Tabitha Freeman, Joanna Lysons, Sarah Foley

**Affiliations:** 1 Centre for Family Research, University of Cambridge; 2 Thomas Coram Research Unit, Department of Social Science, UCL Institute of Education; 3 Centre for Family Research, University of Cambridge

**Keywords:** solo mothers, single mothers by choice, parenting, child adjustment

## Abstract

Findings are presented of the second phase of a longitudinal study of families created by single mothers by choice. Forty-four single heterosexual mothers were compared with 37 partnered heterosexual mothers, all with a donor-conceived child aged around 8–10 years. Standardized interview, observational, and questionnaire measures of maternal wellbeing, mother-child relationships and child adjustment were administered to mothers, children, and teachers. There were no differences in maternal mental health, the quality of mother-child relationships or children’s emotional and behavioral problems between family types. However, higher levels of parenting stress and higher levels of children’s prior adjustment difficulties were each associated with children’s adjustment difficulties in middle childhood irrespective of family type. The findings suggest that the presence of two parents—or of a male parent—is not essential for children to flourish, and add to the growing body of evidence that family structure is less influential in children’s adjustment than the quality of family relationships.

Since the 1980s, a growing number of single heterosexual women have made an active decision to parent alone and have had children through donor insemination. These women are often described as “single mothers by choice” (Bock, 2000; Hertz, 2006), although many have reported that, due to the absence of a partner and their increasing age, they did not have a choice if they wished to become parents (Graham & Braverman, 2012; Graham, 2014; Jadva, Badger, Morrissette, & Golombok, 2009; Murray & Golombok, 2005a). In 2000, Australia was one of the first countries to enact legislation allowing single women to use assisted reproduction procedures such as sperm donation. However, a survey conducted at the time found that only 38% of the population were in favor of single women having children in this way (Kovacs, Morgan, Wood, Howlett, & Forbes, 2003). Criticism of single mothers by choice reached its peak in the United States in 2009, when a single woman gave birth to octuplets following donor insemination (Garrison, Yoshino, & Ho, 2009; Pennings, Klitzman, & Zegers-Hochschild, 2016). In the United Kingdom, a change in the law in 2008 that no longer required fertility clinics to take account of the child’s need for a father, but instead to consider the child’s need for supportive parenting, sparked controversy in the British Parliament (McCandless & Sheldon, 2010) and media (Zadeh & Foster, 2016). The original law meant that most fertility clinics only offered treatment to couples who would create traditional families; the new legislation opened the door to single women.

A primary reason for the opposition to single women accessing assisted reproductive procedures such as donor insemination, was the belief that fathers are essential for children’s psychological adjustment (Silverstein & Auerbach, 1999; Biblarz & Stacey, 2010). Two sets of studies have addressed the question of whether children without fathers are more at risk for psychological problems than children in traditional families, although it is important to note that any effects of the absence of a male parent are confounded by the absence of a second parent irrespective of that parent’s gender. The first set of studies, which focused on children raised in single mother families following their parents’ divorce, was initiated in response to the increasing divorce rates from the 1970s onward, and included investigations conducted in the United States (Hetherington, Cox, & Cox, 1982; Hetherington, 1988; Hetherington & Stanley-Hagan, 1999), the United Kingdom (Dunn, Deater-Deckard, Pickering, O’Connor, Golding, & the ALSPAC Study Team, 1998; Dunn, Davies, O’Connor, Sturgess, 2001; Dunn, 2008), and Australia (Pryor & Rodgers, 2001). It was found that the children were, on average, more likely to experience emotional and behavioral difficulties, and less likely to perform well at school, than children with both parents at home. However, many children whose parents divorced did not show negative effects, and many of those who did, improved over time, especially if the divorce resulted in a more amicable relationship between their parents (Amato, 2000, 2001, 2005; Hetherington & Stanley-Hagan, 1999; Dunn, 2008; Coleman & Glenn, 2009).

The second set of studies focused on children of unmarried single mothers, many of whom had unplanned pregnancies. These studies were prompted by the sharp rise in the number of children born to unmarried mothers from the 1960s, when the rate was under five per cent, to the early 2000s, when it rose to 20% in the United States (McLanahan, 2012) and 15% in the United Kingdom (Kiernan, 2006). Two of the most highly regarded investigations are the Fragile Families Study in the United States, which included children born between 1998 and 2000 (Reichman, Teitler, & McLanahan, 2001; Waldfogel, Craigie, & Brooks-Gunn, 2010), and the Millennium Cohort Study in the United Kingdom, which examined children born around 2000 (Hansen, Johnson, Joshi, et al., 2008; Kiernan & Mensah, 2010). Like the children with divorced single mothers, the children of unmarried single mothers in both studies were found to show more emotional, behavioral, and educational problems than those with married parents.

Nevertheless, the poorer outcomes for children in single mother families than for children with two parents are not necessarily attributable to the absence of a father. Reviews of research on factors associated with adjustment problems among children of divorced parents have concluded that it is the experiences that often accompany single motherhood, rather than single motherhood per se, that appear to be responsible for children’s difficulties. One important predictor is the reduction in income that many single mothers experience following divorce, often necessitating a move to a different neighborhood where the family has no roots or support networks (Amato, 2000, 2005; Hetherington & Stanley-Hagan, 2002; McLanahan & Sandefur, 1994; Pryor & Rodgers, 2001). Another cause of children’s difficulties is witnessing conflict between their parents, sometimes for years before the parents separate (Amato, 2001, 2005; Pryor & Rodgers, 2001; Coleman & Glenn, 2009). In addition, depression is high among recently divorced mothers, which can impair the ability to be an effective parent (Amato, 2000; Dunn et al., 1998; Hetherington & Stanley-Hagan, 2002), and single mothers often lack social support, which can also have an adverse effect on parenting (Taylor & Conger, 2017). Similar factors are at play among children of unmarried single mothers. As with the children of divorced mothers, the poorer outcomes for the children of unmarried single mothers, compared to children with two parents, are largely explained by greater socioeconomic disadvantage and the mothers’ poorer mental health, rather than single parenthood, in itself (Waldfogel et al., 2010; Kiernan & Mensah, 2010).

Much of the concern regarding single mothers by choice stems from the assumption that if children of divorced or unmarried single mothers are more at risk for psychological problems than children with two parents, then children born to single mothers through donor insemination would also experience raised levels of emotional and behavioral difficulties. However, single mothers by choice differ from divorced or unmarried single mothers in ways that may be salient for children’s psychological adjustment; they have made an active decision to parent alone rather than finding themselves in this situation unintentionally, and the financial hardship, parental conflict, maternal depression, and lack of social support associated with adverse outcomes for children in single mother families do not generally apply to children of single mothers by choice. Instead, single mothers by choice are typically well-educated women in professional occupations who become mothers in their late 30s or early 40s, and who have put support arrangements in place before becoming pregnant (Bock, 2000; Hertz, 2006; Jadva et al., 2009; Murray & Golombok, 2005a). Moreover, children born to single mothers by donor insemination have not been separated from a father with whom they may have had a strong bond. Nevertheless, they do face a situation that children from other kinds of single mother families do not; unless they have a known donor, they do not know the identity of their biological father as they are growing up.

The first study to compare heterosexual single mothers by choice with heterosexual married mothers, all with donor-conceived children, found no differences in the mothers’ psychological wellbeing, adaptation to parenthood, warmth, emotional involvement and bonding with their infants. However, the single mothers showed lower levels of interaction and sensitive responding to their infants, possibly because the presence of a partner allowed mothers in two-parent families more time with their babies (Murray & Golombok, 2005a). When the children were aged 2, the single mothers showed greater joy and less anger toward their children than the married mothers (Murray & Golombok, 2005b), as assessed by the Parent Development Interview, an interview technique designed to assess the nature of the emotional bond between the mother and the child (Slade, Belsky, Aber, & Phelps, 1999). In addition, the children with single mothers showed fewer emotional and behavioral problems than those with married mothers. However, 2-year-old children of single mothers by choice are too young to understand the personal and social significance of the absence of a father.

The first phase of the present study examined the quality of mother-child relationships and the psychological adjustment of preschool and early school-age children born to single heterosexual mothers through sperm donation. The families were first seen when the children were aged 5½ years, on average, and compared with a matched comparison group of two-parent heterosexual families whose children had also been conceived by sperm donation (Golombok, Zadeh, Imrie, Smith, & Freeman, 2016). The children had all been conceived using anonymous donors, although some had identifiable donors which means that they are legally entitled to request the identity of their donor on reaching adulthood should they wish to do so (Freeman, Zadeh, Smith, & Golombok, 2016). No differences in parenting quality or child adjustment were found between family types apart from lower mother-child conflict in the solo mother families. However, financial difficulties and parenting stress were associated with raised levels of children’s adjustment problems in both family types.

From the low level of psychological problems shown by the children of single mothers by choice at Phase 1 of the study, it seemed that not knowing the identity of their biological father did not impair their psychological wellbeing. However, the children were too young to fully understand the circumstances of their conception. Although they were aware that they did not have a father and questioned their mothers about the reason for this, they showed little interest in their donor (Zadeh, Freeman, & Golombok, 2017). It is not until middle childhood that children develop an understanding of biological inheritance (Brodzinsky, 2011; Solomon, Johnson, Zaitchik, & Carey, 1996; Williams & Smith, 2010) and grasp what it means to be donor conceived (Blake, Casey, Jadva, & Golombok, 2014; Blake, Casey, Readings, Jadva, & Golombok, 2010). Thus, the aim of this second phase of the study was to examine whether children born through sperm donation to single mothers by choice are at risk for psychological problems following the transition to middle childhood and, if so, to examine the nature of these problems and the mechanisms involved.

The study is grounded within a developmental systems approach (Aldwin, 2014; Overton, 2015), which emphasizes the bidirectional nature of relations between the social environment, parenting and child adjustment. More specifically, the study was guided by the theoretical and research literature on parenting showing that the quality of children’s relationships with their parents is associated with children’s psychological adjustment, such that positive aspects of parenting including warmth, sensitivity, acceptance and parental psychological wellbeing are associated with positive child adjustment whereas conflict, hostility, rejection and parental psychological problems are associated with more negative outcomes for children, with children’s individual characteristics influencing parenting and parental wellbeing (Bornstein, 2019; Collins, Maccoby, Steinberg, Hetherington, & Bornstein, 2000; Lamb, 2012). In line with that framework and the findings of Phase 1 of the study, it was hypothesized that children’s adjustment difficulties would not differ according to the number of parents in the family, but instead would be associated with parenting quality including maternal mental health problems, financial difficulties, and children’s preexisting emotional and behavioral problems.

## Method

### Participants

At the first phase of the study, parents were asked for permission to contact them again for follow up and all agreed (see Golombok et al., 2016 for details of the initial recruitment of families to the study). The parents were approached by telephone, letter or e-mail when the children were aged around 8–10 years. The current study involved 44 single heterosexual mothers and their donor-conceived children (22 boys and 22 girls), and 37 partnered heterosexual mothers and their donor-conceived children (22 boys and 15 girls), representing 78.6% of the families who participated at Phase 1. Of the 22 families who were lost to follow up, 12 could not be traced (6 (12%) single mother families and 6 (12%) two-parent families) and 10 declined to participate (1 (2%) single mother family and 9 (17%) two-parent families). Excluding the 12 families who could not be traced, the participation rate was 89%. There was no significant difference between family types in the proportion of families from Phase 1 who participated in Phase 2.

As illustrated in Table 1, there were no differences between family types in the age or gender of the children. However, the children in single mother families were less likely to have siblings than those in two-parent families, χ^2^(1) = 9.66, *p* = .008, Cramér’s *V* = .36. The age of the mothers differed significantly between family types, reflecting the older age of the single (*M*_age_ = 48.59, *SD* = 3.56) than the partnered mothers (*M*_age_ = 44.16, *SD* = 3.56), *t*(79) = 5.58, *p* < .001, Cohen’s *d* = 1.24. The mothers did not differ in terms of other key demographic characteristics, such as working status, education, ethnicity, perceived financial difficulties, and prior psychiatric contact. None of the single mothers was married or cohabiting at Phase 2. Since Phase 1, one couple in the comparison group had divorced, and another couple had separated.[Table-anchor tbl1]

### Procedure

Researchers trained in the study techniques visited the families in their homes throughout the United Kingdom. Written informed consent for the mothers and children to participate in the investigation was obtained from the mothers, and the children gave verbal assent. Ethical approval for the study was granted by the University of Cambridge Psychology Research Ethics Committee. The mothers were administered a standardized interview that was audio recorded. The mothers and children also completed standardized questionnaires and participated together in a video-recorded observational task that lasted approximately 10 min. Following written permission from the mothers, the children’s teachers were asked to complete a questionnaire to give an independent assessment of the children’s adjustment. The teachers were assured that their responses to the questionnaire would not be reported back to the child’s family or school, and written informed consent was obtained from the teachers. As data were obtained by interview on issues relating to the children’s families, it was not possible for the researchers to be “blind” to family type. However, the section of the interview on the children’s emotional and behavioral problems was rated by a child psychiatrist who was unaware of the children’s family type.

### Measures

#### Parenting interview

The mothers were interviewed using an adaptation of a semistructured interview designed to assess the quality of mother-child relationships. The interview has been validated against observational ratings of mother-child relationships in the home (Quinton & Rutter, 1988), and has been used successfully in previous studies of donor-conception families with school-age children (Golombok, Ilioi, Blake, Roman, & Jadva, 2017). Detailed accounts are obtained of the child’s behavior and the mother’s response to it, with particular attention to interactions relating to warmth and control. A flexible style of questioning is used to elicit sufficient information for each variable to be rated by the interviewer according to a standardized coding scheme based upon a detailed coding manual. Thus, ratings are made by the researcher using in-depth information obtained from the mother rather than by the mother herself.

The following interview variables were coded (a) *expressed warmth* from 0 (*none*) to 5 (*high*) took account of the mother’s tone of voice, facial expressions and gestures in addition to what the mother said about the child; (b) *mother-to-child warmth* represented the frequency and spontaneity of affection shown by the mother to the child; (c) *child-to-mother warmth* from 0 (*none*) to 3 (*marked*) represented the frequency and spontaneity of affection shown by the child to the mother; (d) *mother’s enjoyment of play* from 1 (*little or none*) to 4 (*a great deal*) assessed the extent to which the mother enjoyed playing with the child; (e) *amount of interaction* from 1 (*little*) to 3 (*high*) assessed the amount of time the mother and child spent in shared activities; (f) *quality of interaction* from 0 (*very poor*) to 4 (*very good*) was based on the extent to which the mother and child wanted to be with each other and enjoyed each other’s company; (g) *conflict* from 0 (*little or none*) to 3 (*a great deal*) measured the extent of disagreement between mothers and their children; (h) *frequency of battles* from 0 (*never/rarely*) to 5 (*daily*) assessed the frequency of mother-child conflict; (i) *level of battles* from 0 (*none*) to 3 (*major***)** assessed the severity of mother-child conflict; and (j) *criticism* from 0 (*none*) to 4 (*considerable*) was based on the amount of criticism of the child by the mother. The interrater reliabilities calculated using (intraclass correlation coefficients) were as follows: *expressed warmth* (.87), *mother-to-child warmth* (.65), *child-to-mother warmth* (.71), *mother’s enjoyment of play* (.76), *amount of interaction* (.74), *quality of interaction* (.93), *conflict* (.86), *frequency of battles* (.97), *level of battles* (.88) and *criticism* (.85).

#### Parental acceptance

The 24-item short form of the Parental Acceptance and Rejection Questionnaire (PARQ; Rohner & Khaleque, 2005) was administered to mothers and children separately to provide total scores of parental acceptance/rejection comprising the subscales of warmth/affection, hostility/aggression, indifference/neglect, and undifferentiated rejection. The mothers completed the questionnaire regarding their feelings toward their children, and the children completed the questionnaire regarding their perceptions of their mothers’ feelings toward them. Higher scores represent greater rejection and lower scores represent greater acceptance, with scores above 60 representing higher levels of rejection than acceptance. A meta-analysis of the reliability of the PARQ (Khaleque & Rohner, 2002) utilized data from 51 studies worldwide and found that internal consistency exceeded .70 in all groups studied. Convergent and discriminant validity have also been demonstrated (Rohner & Khaleque, 2005). In the current study, the PARQ showed high internal consistency (α = .77 for mothers and α = .88 for children).

#### Parent-child interaction

The Etch-A-Sketch task (Stevenson-Hinde & Shouldice, 1995) was used to obtain an observational assessment of the quality of interaction between the mother and child. The Etch-A-Sketch is a drawing tool with two dials that allow one person to draw vertically and the other to draw horizontally. The mother and child were asked to copy a picture of a boat, each using one dial only, with clear instructions not to use the other dial. The sessions were video-recorded and scored using the Parent Child Interaction Rating Coding Scheme (PARCHISY: Deater-Deckard & Petrill, 2004) by a researcher who was blind to family type to assess the construct of mutuality; that is, the extent to which the mother and child engaged in positive dyadic interaction characterized by warmth, mutual responsiveness, and cooperation. The following variables were rated on a 7-point scale ranging from 1 (*no instances*) to 7 (*constant, throughout the interaction*): (a) *child’s responsiveness to mother* assessed the extent to which the child responded immediately and contingently to the mother’s comments, questions or behaviors; (b) *mother’s responsiveness to child* assessed the extent to which the mother responded immediately and contingently to the child’s comments, questions or behaviors; (c) *dyadic reciprocity* assessed the degree to which the dyad showed shared positive affect, eye-contact and a ‘turn-taking’ (conversation like) quality of interaction; and (d) *dyadic cooperation* assessed the degree of agreement about whether and how to complete the task. The interrater reliabilities (intraclass correlation coefficients) were as follows: *mother responsiveness* (.87), *child responsiveness* (.83), *dyadic reciprocity* (.70) and *dyadic cooperation* (.68).

#### Maternal mental health

The Trait Anxiety Inventory (TAI: Spielberger, 1983), the Edinburgh Depression Scale (EDS: Thorpe, 1993) and the short form of the Parenting Stress Index (PSI: Abidin, 1990) were completed by mothers to assess anxiety, depression and stress associated with parenting, respectively. Each of these instruments, for which higher scores represent greater difficulties, has been shown to have good reliability and to discriminate well between clinical and nonclinical groups. In the present study, all three scales showed excellent internal consistency (EDS α = .86; TAI α = .91; PSI α = .90).

#### Strengths and Difficulties Questionnaire

The presence of child adjustment problems was assessed with the Strengths and Difficulties Questionnaire (SDQ: Goodman, 2001) administered to mothers. This questionnaire asks parents to reflect on their child’s behavior over the past 6 months and rate whether an item is *not true, somewhat true,* or *certainly true* for their child. The SDQ produces a total score of children’s adjustment difficulties, with scores of 14 or above classified as indicative of psychiatric disorder. An independent assessment of the children’s psychological adjustment was obtained from teachers. For teachers’ questionnaires, scores of 12 or above are classified as indicative of psychiatric disorder.

The SDQ has been shown to have good internal consistency, test-retest and interrater reliability, and concurrent and discriminative validity (Goodman, 2001). For example, based on an epidemiological sample of more than 10,000 children in the U.K. (Goodman, 2001), internal consistency (Cronbach’s alpha) was found to be 0.73, test–retest reliability after 4–6 months was 0.62 and, in terms of validity, scores above the 90th centile predicted a substantially raised probability of independently diagnosed psychiatric disorders. In a review of the reliability and validity of the SDQ based upon 48 studies involving more than 130,000 children, Stone, Otten, Engels, Vermulst, & Janssens (2010) found the psychometric properties of the SDQ to be strong. Internal consistencies for mothers and teachers, respectively, in the current study were α = .86 and α = .83.

#### Ratings of psychiatric disorder

The presence of child psychiatric disorder was assessed during the interview with the mother using a standardized procedure (Rutter, Cox, Tupling, Berger, & Yule, 1975). Detailed descriptions were obtained of any emotional, behavioral, or developmental problems shown by the child. These descriptions of actual behavior, which included information about where the behavior was shown, severity of the behavior, frequency, precipitants, and course of the behavior over the past year, were transcribed verbatim and rated by a child psychiatrist who was unaware of the nature of the study. A high level of reliability (*r* = .85) between ratings made by social scientists and those made “blindly” by a child psychiatrist has been demonstrated for this procedure, and validity has been established through a high level of agreement between interview ratings of children’s psychological problems and mothers’ assessments of whether or not their children had emotional or behavioral difficulties (Rutter et al., 1975). Psychiatric disorder, when identified, was rated according to severity on a 3-point scale ranging from 0 (*no disorder*), 1 (*slight but definite*) to 2 (*definite or marked*), and type (emotional disorder, conduct disorder, mixed emotional and conduct disorder, developmental disorder, Attention Deficit Hyperactivity Disorder, psychotic disorder, and other disorder).

### Analysis Plan

Prior to addressing the research questions, we established that the factor structure of the parenting interview variables at Phase 1 was replicated at Phase 2 using principal components analysis. Two factors, with a mean factor loading of 0.73, explained 58.64% of the variance. The first factor, reflecting positive parenting, explained 35.21% of the variance and included *expressed warmth* (0.67), *mother-to-child warmth* (0.89), *child-to-mother warmth* (0.78), *mother’s enjoyment of play* (0.68), *amount of interaction* (0.80) and *quality of interaction* (0.68). The second factor, reflecting negative parenting, explained 23.43% of the variance, and included *conflict* (0.82), *frequency of battles* (0.78), *level of battles* (0.46) and *criticism* (0.74). The factor loadings at Phase 2 were similar to those of Phase 1: *expressed warmth* (0.74), *mother-to-child warmth* (0.73), *child-to-mother warmth* (0.62), *mother’s enjoyment of play* (0.62), *amount of interaction* (0.66), *quality of interaction* (0.66), *conflict* (0.83), *frequency of battles* (0.70), *level of battles* (0.63) and *criticism* (0.62).

Following this, we used MANCOVAs to examine whether there were group differences between single mother versus two-parent families in terms of the quality of mother-child relationships, maternal mental health and child adjustment. For each MANCOVA, we covaried the demographic variables that differed by family type, that is, parent age and number of siblings. Next, we used partial correlations to explore associations between the family process and child adjustment variables while controlling for prior levels of the variables at Phase 1. We then built a linear regression model in Mplus (Muthén & Muthén, 2012) to examine the independent contribution of the Phase 2 family process variables on child adjustment scores, over and above the stability of child adjustment scores from Phase 1. A full information approach was used so that all cases with data at either time point could be used in the analyses. This approach is suitable for regression models and produces less biased estimates than traditional missing data handling procedures (Enders, 2001).

## Results

### Parenting

As illustrated in Table 2, a MANCOVA was carried out for the interview variables comprising the positive parenting factor (*expressed warmth*, *mother-to-child warmth*, *child-to-mother warmth*, *mother’s enjoyment of play*, *amount of interaction*, and *quality of interaction*), with family type (one-parent vs. two-parent) as a between-subjects factor, and parent age and number of siblings included as covariates. Wilks’ λ was not significant, *F*(6, 67) = 0.96, *p* = .463, η^2^ = .08, demonstrating no differences in the magnitude of positive parenting between the single mother and two-parent families.[Table-anchor tbl2]

Similarly, a MANCOVA, with family type (one-parent vs. two-parent) as a between-subjects factor, and parent age and number of siblings as covariates, was carried out for the interview variables that contributed to the negative parenting factor (*conflict*, *frequency of battles*, *level of battles* and *criticism*). Wilks’ λ was not significant, *F*(4, 65) = 1.83, *p* = .134, η^2^ = .10, showing no differences in the level of negative parenting between the single mother and two-parent families.

A MANCOVA with family type (one-parent vs. two-parent) as a between-subjects factor, and parent age and number of siblings as covariates, was also carried out for parental acceptance/rejection (mother and child reports). Wilks’ λ was not significant, *F*(2, 66) = 1.09, *p* = .343, η^2^ = .03, showing no difference in levels of acceptance/rejection between the single mother and two-parent families.

The measures relating to parent–child mutuality were also examined. A MANCOVA was carried out for the four mutuality variables (*mother responsiveness*, *child responsiveness*, *dyadic reciprocity* and *dyadic cooperation*), with family type (one-parent vs. two-parent) as a between-subjects factor, and parent age and number of siblings as covariates. Wilks’ λ was not significant, *F*(4, 58) = .27, *p* = .894, η^2^ = .02, demonstrating no differences in mother-child interaction between the single mother and two-parent families.

As shown in Table 2, a MANCOVA with family type (one-parent vs. two-parent) as a between-subjects factor, and parent age and number of siblings included as covariates, was carried out for the maternal mental health variables (depression, anxiety and parenting stress). Wilks’ λ was not significant, *F*(3, 73) = 1.08, *p* = .361, η^2^ = .04, showing no differences in mothers’ mental health problems between the two family types.

### Child Adjustment

An ANCOVA was carried out for children’s SDQ scores as rated by mothers, with family type (one-parent vs. two-parent) as a between-subjects factor, and parent age and number of siblings as covariates. The analysis showed no difference in child adjustment between family types, *F*(1, 76) = .03, *p* = .875, η^2^ = .00.

In light of the teachers’ response rate (70% of the children had teacher questionnaires), a separate ANCOVA, with family type (one-parent vs. two-parent) as a between-subjects factor, and parent age and number of siblings as covariates, was conducted for the teachers’ SDQ scores. The analysis showed a significant but modest group difference in child adjustment, *F*(1, 53) = 4.28, *p* = .043, η^2^ = .08, such that children from single-mother families received higher scores (*n* = 29, *M* = 7.10, *SD* = 5.18) than children from two-parent families (*n* = 28, *M* = 5.29, *SD* = 4.57).

There were no group differences in the number of children scoring above the cut-off on the SDQ for either mothers’ ratings (*n* = 6 and *n* = 6), χ^2^(1) = .08, *p* = .777, or for teachers’ ratings, (*n* = 7 and *n* = 4), χ^2^(1) = .77, *p* = .380, demonstrating no differences in the number of children with scores indicative of psychiatric disorder between the single mother and two-parent families.

According to the independent psychiatric ratings, the majority of the sample (89%) had no problems. There was no difference between family types in the proportion of children who displayed a psychiatric disorder; s*light n* = 2 and *marked n* = 1 in the single mother families, and *slight n* = 4 and *marked n* = 1 in the two-parent families, Fisher’s exact = .70, *p* = .554. Of these children, one showed emotional problems, one showed conduct problems, one had developmental problems and five exhibited multiple problems, such as a mixture of emotional and conduct problems, or a combination of developmental and conduct problems.

### Predictors of Child Adjustment

After establishing that there was no difference in child adjustment between family types according to mothers’ SDQ scores, associations between the family process variables and the children’s SDQ scores were examined (see Table 3). To examine the specificity of the associations, partial correlations were conducted controlling for prior levels of the scores (Phase 1 scores were available for all measures apart from the child PARQ). Mothers’ SDQ scores were significantly associated with parenting stress (*r* = .47*), the negative parenting factor derived from the interview (*r* = .38*) and children’s reports of parental acceptance/rejection (*r* = .31*).[Table-anchor tbl3]

A multiple linear regression analysis was carried out to examine the independent contribution of the family process variables that were significantly correlated with the children’s SDQ scores, over and above the stability of children’s difficulties as assessed by mother’s SDQ scores at Phase 1 of the study. Specifically, the children’s SDQ scores at Phase 2 were regressed on to parenting stress, children’s reports of parental acceptance/rejection, the interview factor reflecting negative parenting and children’s SDQ scores at Phase 1. This just-identified model explained significant variance in children’s SDQ scores at Phase 2, *R*^2^ = 0.51, *SE* = 0.08, z = 6.47, *p* < .001. As illustrated in Table 4, prior levels of difficulties, β = −0.46, *SE* = 0.09, *p* < .001, and higher levels of parenting stress, β = 0.29, *SE* = 0.10, *p* = .003, predicted unique variance in children’s SDQ scores at Phase 2, showing that children’s adjustment difficulties in middle childhood were not only associated with their adjustment difficulties in early childhood, but also with their mothers’ parenting stress.[Table-anchor tbl4]

## Discussion

In line with the findings at Phase 1 of the study when the children were in their preschool or early school years, no differences were identified between the single mother families and the two-parent families in parenting or child adjustment when the children reached middle childhood. With respect to parenting, the family types were similar in terms of the quality of mother-child relationships as assessed by standardized interview, the quality of mother-child interaction as assessed by an observational measure, and parental acceptance as measured by questionnaire. In addition, there were no differences between the single and partnered mothers in anxiety, depression, or stress associated with parenting, and the mothers’ mean scores for anxiety and depression were below the cut-off points of 40 (Grant, McMahon, & Austin, 2008) and 13 (Matthey, Henshaw, Elliott, & Barnett, 2006), respectively, based on normative data. Thus, using multiple methods involving both representational and behavioral measures of parent–child relationships (Imrie, Jadva, Fishel, & Golombok, 2019) with both mothers and children, and standardized measures of mothers’ mental health, it appeared that families formed by single mothers by choice were functioning as well as families with two parents when their children were around nine years old.

There was a similar pattern of findings for child adjustment. The children of single mothers by choice did not differ from children with two parents in terms of their scores on the Strengths and Difficulties Questionnaire as completed by mothers. It should also be noted that the average scores were in line with the population norms of 8.4 and 6.6 for the parent and teacher SDQ, respectively (Meltzer, Gatward, Goodman, & Ford, 2000). Moreover, the number of children with scores above the cut-off point for psychiatric disorder did not differ by family type (*n* = 6 and *n* = 6, respectively). The teachers’ scores on the Strengths and Difficulties Questionnaire did differ between family types, with the children of single mothers obtaining significantly higher scores than those in two-parent families. However, the teachers’ scores were within the normative range, suggesting that the children in both family types were generally well adjusted. It should also be noted that the significant finding for teachers’ SDQ scores may have resulted from chance, given the large number of group comparisons. In terms of the number of children with teachers’ scores above the cut-off for psychiatric disorder, there was no difference between the children with one and two parents, and the proportion scoring above cut-off in both family types was small (*n* = 7 and *n* = 4, respectively), reflecting a smaller percentage in the problematic range compared to general population norms (Meltzer et al., 2000). In addition, the independent psychiatrists’ ratings suggested that the children were functioning well, with only one child in each family type displaying severely problematic behavior. It seems, therefore, that even when donor-conceived children of single mothers reach the age at which they understand the significance of not having a father, they are no more likely to show adjustment difficulties than children who grow up with a father.

Although there was little difference in parenting or child adjustment between family types, in line with a developmental systems conceptual framework (Aldwin, 2014; Overton, 2015), higher levels of parenting stress and higher levels of children’s prior adjustment difficulties were each associated with children’s adjustment difficulties in middle childhood irrespective of family type. The finding for parenting stress replicates that of the first phase of the study (Golombok et al., 2016) and supports the hypothesis that children’s emotional and behavioral problems would be associated with maternal mental health problems. Similarly, the association between children’s adjustment difficulties at Phase 1 and Phase 2 of the study is consistent with the hypothesis that adjustment difficulties in middle childhood would be associated with children’s preexisting emotional and behavioral problems. The lack of association between either maternal anxiety or depression and children’s adjustment difficulties appears to reflect the low levels of anxiety and depression in the sample. For example, only five women’s ratings of depression fell within the problematic range on the Edinburgh Depression Scale. Our findings highlight the value of considering multiple components of parents’ wellbeing, everyday stressors, rather than clinically relevant symptoms of anxiety and depression, contributed to children’s adjustment difficulties.

Interestingly, in contrast to the findings reported at Phase 1 (Golombok et al., 2016), financial difficulties did not appear to contribute to children’s adjustment problems at Phase 2. Time-related characteristics may help explain this null effect, specifically the lack of stability in financial difficulties and the differences in working patterns from Phases 1 to 2, with 16 mothers entering the workforce during this period. The small minority of families who experienced definite financial difficulties at Phase 2 is consistent with previous research showing that single mothers by choice are generally financially secure (Bock, 2000; Graham & Braverman, 2012; Jadva et al., 2009), and may explain why financial difficulties were not associated with children’s adjustment problems.

Studying the adjustment of donor-conceived children born to single mothers by choice is not only of interest in its own right as little is known about the development and wellbeing of children in this new family form, but also is of theoretical interest as it enables the effects of growing up in a single mother family to be investigated without the potentially confounding effects of parental conflict, financial difficulties and maternal mental health problems. The similarities in parenting and child adjustment between children in one-parent and two-parent families in the present study are in direct contrast to the findings of studies of families headed by divorced or unmarried single mothers, which found higher levels of children’s emotional and behavioral problems compared to children in two-parent families. This discrepancy may be attributable to the differing social circumstances of single mothers by choice, who made an active decision to parent alone and planned their lives accordingly, and divorced and unmarried single mothers, who found themselves in this situation unintentionally. The findings of the present study thus add weight to the view that the raised levels of adjustment problems shown by children of divorced and unmarried single mothers result from the adverse circumstances that often accompany single motherhood, rather than single motherhood, in itself.

A limitation of the study is the modest sample size, which may have resulted in differences between the single mother and two-parent families not being detected. The relatively low intraclass correlation coefficients for mother-to-child warmth and child-to-mother warmth are likely to have resulted from the lack of information on nonverbal aspects of warmth, such as facial expressions, that were taken into account in the ratings made by the interviewer, but were unavailable to the second rater who was coding from audio recordings. A further limitation is that participants were originally recruited through a private fertility clinic, and thus the findings may not reflect the experiences of parents and children in families formed by single mothers by choice using other routes, such as sexual intercourse or online connection sites (Jadva, Freeman, Tranfield, & Golombok, 2018), about whose socioeconomic circumstances little is yet known.

An important advantage of the study was that the children in the comparison group of two-parent families had all been conceived by donor insemination thus controlling for the use of donor insemination by the single mothers by choice. In addition, a high retention rate was obtained at Phase 2, and the use of similar measures at both phases of the study enabled the Phase 1 scores to be controlled for in the Phase 2 analysis. A further advantage was that the families were recruited when the children were young, thus avoiding sample bias resulting from children who were distressed about their family declining to join the study.

With the exception of the investigation by Chan, Raboy, and Patterson (1998), which focused primarily on single lesbian mothers and produced similar findings to the present investigation, this is the only study of parenting and child adjustment in families formed by single mothers by choice when the children reach middle childhood. Although, by this age, children have acquired a more sophisticated understanding of what it means to be conceived by donor insemination to a single mother and not know the identity of their biological father, they continued to show positive relationships with their mothers and high levels of psychological adjustment. This suggests that the presence of two parents—or of a male parent—is not essential for children to flourish, thus adding to the growing body of evidence (Golombok, 2015; Lamb, 2012; Patterson, 2009) that family structure is less influential in children’s adjustment than the quality of family relationships.

## Figures and Tables

**Table 1 tbl1:** Means, Standard Deviations, and Differences in Sociodemographic Information by Family Type

	Solo mothers		Partnered mothers			
Demographic characteristics	*M*	*SD*	*M*	*SD*	*t*	*p*
Age of mother (years)	48.59	3.56	44.16	3.56	5.58	<.001
Age of child (years)	9.45	1.61	9.05	1.22	1.24	.218
	*n*	%	*n*	%	χ^2^	*p*
Child gender					.73	.395
Male	22	50	22	59.5		
Female	22	50	15	40.5		
Siblings					9.66	.008
None	20	45.5	9	24.3		
One	15	34.1	21	56.8		
Two or more	1	2.3	7	18.9		
Mother’s working status					4.47	.107
Not working	5	11.4	8	21.6		
Part-time	16	36.4	18	48.6		
Full-time	23	52.3	11	29.7		
Perceived financial difficulties					3.14	.370
None	34	77.3	30	81.1		
Minor	8	18.2	3	8.1		
Definite	2	4.5	4	10.8		
Mother’s education					3.46	.178
Below university degree	12	27.3	14	37.8		
Undergraduate degree	14	31.8	15	40.5		
Postgraduate degree	18	40.9	8	21.6		
Mother’s ethnicity						
White British	41		34		.40	.528
BAME	3		3			
Mother’s psychiatric contact					3.58	.167
None	40	90.9	29	78.4		
General practitioner	4	9.1	6	16.2		
Outpatient	0	0	2	5.4		
*Note*. BAME = Black Asian Minority Ethnic.						

**Table 2 tbl2:** Means, Standard Deviations, and Differences in Parenting, Mother’s Psychological Wellbeing, and Mutuality by Family Type

	Solo mothers		Partnered mothers				
Family functioning	*M*	*SD*	*M*	*SD*	*F*	*p*	η^2^
Child adjustment							
Mother SDQ^a^	8.40	6.31	9.03	5.86	.03	.875	.00
Teacher SDQ^b^	7.10	5.18	5.29	4.57	4.28	.043	.08
Positive parenting^c^							
Expressed warmth	3.87	.92	3.95	1.05	.16	.695	.00
Mother-to-child warmth	2.44	.60	2.57	.60	.73	.397	.01
Child-to-mother warmth	2.33	.66	2.46	.65	.46	.499	.01
Mother’s enjoyment of play	3.28	.65	3.05	.82	1.88	.175	.03
Amount of interaction	2.33	.58	2.43	.69	.20	.660	.00
Quality of interaction	2.97	.60	2.95	.82	.02	.882	.00
Negative parenting^c^							
Conflict	1.18	.55	1.06	.67	2.18	.144	.03
Frequency of battles	2.43	.15	2.19	1.71	.57	.465	.01
Level of battles	1.55	.55	1.31	.59	1.76	.190	.03
Criticism	1.43	.75	1.75	1.02	1.23	.271	.02
Parental acceptance/rejection^a^	36.00	8.92	37.84	10.18	.59	.447	.01
Parental acceptance/rejection^d^	30.00	5.13	31.42	5.24	.98	.325	.01
Mutuality^e^							
Child responsiveness	4.91	.87	4.97	.66	.07	.786	.00
Mother responsiveness	4.97	.97	5.19	.60	.37	.545	.01
Dyadic reciprocity	3.00	.85	3.03	.75	.01	.942	.00
Dyadic cooperation	2.97	1.06	3.06	1.00	.04	.852	.00
Psychological wellbeing^a^							
Trait Anxiety Inventory	35.48	7.09	38.11	10.55	.07	.790	.00
Edinburgh Depression Scale	5.07	3.76	6.35	4.82	2.01	.160	.03
Parenting Stress Index	62.53	13.19	67.51	17.48	.08	.777	.00
*Note*. SDQ = Strengths and Difficulties Questionnaire.							
^a^ parent report. ^b^ teacher report. ^c^ parental interview. ^d^ child report. ^e^ observation.							

**Table 3 tbl3:** Correlations Between Main Study Variables

Study variables	1	2	3	4	5	6	7	8	9	10	11	12	13	14	15	16
1. Child difficulties^a^	—															
2. Child age	−.00	—														
3. Child gender	−.14	−.16	—													
4. Financial difficulties	.20	−.12	.10	—												
5. Positive parenting^b^	.14	−.05	.05	−.10	—											
6. Negative parenting^b^	.39**	−.06	−.18	.14	.00	—										
7. Parental rejection^a^	.31**	.08	−.11	.06	−.33**	.29*	—									
8. Parental rejection^c^	.31**	−.16	−.18	.06	−.25	.27*	.36*	—								
9. Parent responsiveness^d^	.09	.17	.03	.22	.20	−.21	−.10	.05	—							
10. Child responsiveness^d^	−.03	.23	.03	.07	−.25	−.01	.14	−.01	.36*	—						
11. Dyadic reciprocity^d^	.02	.14	.02	.13	.09	.06	.15	.06	.19	.15	—					
12. Dyadic co-operation^d^	−.09	.17	.11	.12	.06	−.19	.01	−.18	.56**	.42**	.19	—				
13. Parent anxiety^a^	.35**	−.14	−.17	.29**	−.14	.31**	.34*	.22	−.06	.05	.06	−.03	—			
14. Parent depression^a^	.25*	−.13	−.01	.34**	−.15	.30*	.25*	.12	−.08	.07	.05	.05	.68**	—		
15. Parenting stress^a^	.56**	−.04	−.12	.40**	−.14	.50**	.46**	.27*	−.01	.03	.02	−.00	.60**	.38**	—	
16. Phase 1 child difficulties^a^	.60**	.10	−.30**	.19	.15	.23	.23*	.23	.03	−.21	−.17	−.12	.31**	.04	.39**	—
^a^ parent report. ^b^ parental interview. ^c^ child report. ^d^ observation.																
* *p* < .05. ** *p* < .01.																

**Table 4 tbl4:** Model Parameter Estimates

	Phase 2 total child difficulties		
Predictor variables	Est.	*SE*	Std. Est.
Parenting stress^a^	.12	.04	.29**
Negative parenting^b^	.54	.61	.09
Parental rejection^c^	.07	.06	.10
Phase 1 child difficulties	.63	.12	.46***
^a^ parent report. ^b^ Negative parenting = factor from parent interview. ^c^ child report.			
** *p* < .01. *** *p* < .001.			
